# Reply to M López-Moreno

**DOI:** 10.1016/j.cdnut.2026.109536

**Published:** 2026-09-22

**Authors:** Saba Vaezi, Bruna O de Vargas, Moul Dey

**Affiliations:** School of Health and Human Sciences, South Dakota State University, Brookings, SD, United States

Dear Editor:

López-Moreno [1] raise important considerations regarding the design and interpretation of findings from the Protein-Distinct Macronutrient-Equivalent Diet (PRODMED) randomized controlled crossover feeding trial [2]. The comments address our published methodology [3] and trial outcomes [2], focusing on the extent of dietary matching between the intervention arms and the reporting framework for prespecified primary outcomes. We welcome the opportunity to clarify these points.

The PRODMED trial was prospectively designed to evaluate minimally-processed pork and lentils (representing the pulse food group) as distinct primary protein sources within plant-forward dietary patterns aligned with healthy dietary guidance [[2], [3], [4], [5]]. The objective was not to evaluate pork and pulses as unrestricted whole-food comparators. Rather, the intended contrast centered on protein-specific attributes, including amino acid profile and protein quality. Approximately 45% of total dietary protein was provided by the assigned primary protein source in each intervention, whereas total protein and selected background dietary characteristics were standardized to maintain a comparable dietary context. Similar approaches have been used in controlled feeding randomized controlled trials, including our own prior trials [[6], [7], [8], [9]].

Once the primary protein target was established, the surrounding diets were adjusted because equivalent protein from pork and pulses inherently differed in their accompanying energy and nutrient contributions. The diets were therefore modified to maintain comparable total energy and protein while remaining within a guideline-aligned, plant-forward framework and a broadly similar whole-food dietary matrix. Fiber provides a useful example: although both plant-forward diets were relatively high in dietary fiber, the inclusion of pulses further increased fiber content. Achieving comparable fiber intake in the pork-based diet therefore required the addition of soluble fiber sources. Similar adjustments were necessary for other nutrients, and because some of these modifications also affected protein intake, greater amounts of eggs and milk were included in the pulse-based diet to maintain comparable total protein across interventions.

A related consideration was how the primary protein target was achieved in the pulse-based diet. Pea protein and plant-based meat products were counted toward the pulse-based primary protein allocation. Matching the protein provided by 162 g/d of pork using intact pulses alone would have required quantities that displaced much of the surrounding diet. Their contribution was modest, representing ∼12% by weight of the pulse-based protein foods [3].

Importantly, despite this close nutritional matching, the protein-related characteristics central to the comparison remained distinct. Amino acid composition and protein quality differed between the pork and pulse protein sources. The Digestible Indispensable Amino Acid Score was 122.7 for the pork protein source and 82.0 for the pulse protein source, and whole-diet essential amino acid-9 scores also differed [3].

These distinctions were reflected in several physiological outcomes. HDL cholesterol and homocysteine differed between interventions, whereas ferritin, Single-Point Insulin Sensitivity Estimator Index, and glucose changed significantly in only 1 intervention arm. Thus, although dietary matching attenuated some ancillary differences inherent to comparing pork and pulses as whole foods, the interventions remained biologically distinguishable.

To the interpretation of the primary outcomes, homocysteine was 1 of 4 prespecified primary endpoints, along with fasting insulin, ferritin, and chair-stand performance [2]. It was used for the sample-size calculation because it required the largest sample to detect a meaningful change, not because it was assigned greater importance than the other primary outcomes. Accordingly, it was interpreted alongside the other prespecified endpoints.

We reported that homocysteine increased after the pork-based diet but remained unchanged after the pulse-based intervention, resulting in a significant between-diet difference. This finding was described in the Results section and explored further because individual responses appeared to vary. The additional analysis was presented as exploratory and hypothesis-generating, and we noted that a larger, adequately powered study would be needed to determine its clinical significance and reproducibility. The purpose of this analysis was to better understand the observed homocysteine response rather than to give this outcome greater weight than the other primary endpoints.

Overall, we agree with López-Moreno that food-based randomized trials require transparent reporting of which dietary characteristics are standardized compared with allowed to vary, and how these design choices shape both the research question and interpretation of outcomes, an objective we sought to address in our publications. Such transparency is particularly important in controlled feeding trials, which involve complex and sometimes competing design considerations, substantial logistical demands, and resource-intensive implementation that cannot readily be modified post hoc [10]. This rigor is essential because well-executed controlled feeding trials remain among the most powerful approaches for establishing causal relationships between dietary exposures and health outcomes in humans.

Controlled feeding trials inherently navigate a fundamental methodological trade-off: leaving background characteristics unmatched introduces confounding from disparate nutrient and caloric loads, whereas standardizing them narrows the biological contrasts. Unlike pharmacological trials that evaluate concentrated doses of single molecules, dietary interventions must balance these competing priorities based on their specific research question. As López-Moreno notes, a pragmatic, unstandardized substitution of pork with pulses addresses a valid and important causal question; however, this was not the question PRODMED was designed to answer. Finally, although all prespecified primary outcomes were reported and analyzed, we acknowledge that additional discussion of their placement within the broader outcome hierarchy may have provided useful context, although such extensive structural framing was beyond the intended scope of the original manuscript.

We appreciate this opportunity to clarify our trial framework. Examining less standardized protein substitutions in future trials will yield vital complementary evidence across diverse dietary contexts.

## Author contributions

SV, MD: drafted the reply; and all authors: reviewed, edited, and approved the final version.

## Declaration of Generative AI and AI-assisted Technologies in the Writing Process

During the preparation of this manuscript, the authors used ChatGPT (OpenAI) and Claude (Anthropic) to improve sentence structure and grammar. All content was subsequently reviewed and edited by the authors, who take full responsibility for the final version of the manuscript.

## Funding

The authors reported no funding received for this study.

## Conflict of interest

The authors report no conflicts of interest.
